# Hyponatremia Is a Specific Marker of Perforation in Sigmoid Diverticulitis or Appendicitis in Patients Older Than 50 Years

**DOI:** 10.1155/2013/462891

**Published:** 2013-02-13

**Authors:** S. A. Käser, R. Furler, D. C. Evequoz, C. A. Maurer

**Affiliations:** ^1^Department of General, Visceral, Vascular, and Thoracic Surgery, Hospital of Liestal of The University of Basel, Rheinstrasse 26, 4410 Liestal, Switzerland; ^2^Department of General, Visceral and Trauma Surgery, Hospital Center of Oberwallis (SZO), Pflanzettastrasse 6, 3930 Visp, Switzerland; ^3^Division of Cardiology, Department of Internal Medicine, Hospital Center of Oberwallis (SZO), Pflanzettastrasse 6, 3930 Visp, Switzerland

## Abstract

*Introduction*. This study aimed to evaluate symptoms and signs, inflammation markers, electrolytes, and ECG signs of increased vagal tone as markers of colon perforation in sigmoid diverticulitis or appendicitis. *Methods*. The records of all patients older than fifty years (only these had routine ECG done) admitted to our emergency station between January 2008 and December 2010 with sigmoid diverticulitis (*n* = 198, diagnosed by computer tomography) or appendicitis (*n* = 84, diagnosed intraoperatively) were retrospectively evaluated. Pain score, heart rate, blood pressure, and body temperature were assessed at presentation. Before starting infusion therapy, blood was taken to do a blood count and to analyze CRP, the electrolytes, and creatinine levels. Then an ECG was done. *Results*. The perforation rate was 37% (*n* = 103). Body temperature, heart rate, sodium, CRP, and leukocytes correlated significantly with infectious colon perforation. However, only body temperature, CRP, and sodium correlated significantly with infectious colon perforation if compared by logistic regression analysis. The prevalence of hyponatremia (sodium level <136 mmol/L) was 29% in the group with infectious colon perforation and 16% in the group without (*P* = 0.013). *Conclusion*. Hyponatremia is a specific marker of infectious colon perforation in patients older than fifty years.

## 1. Introduction

Older patients with abdominal sepsis present with altered symptoms [1] and their outcome is poorer than in younger patients [2]. As the perforation rate in appendicitis correlates with older age [3], and as sigmoid diverticulitis per se rarely occurs below the age of 50 years [4], infectious colon perforation is believed to be more frequent in older patients than in the young.

CRP is a known strong marker of infectious colon perforation [3, 4]. Hyponatremia (sodium level <  136 mmol/L) is the most frequent electrolyte disorder and it is associated with an increase of morbidity and mortality [5, 6]. It can occur as the consequence of the early systemic inflammatory response probably mediated by IL-6 and vasopressin [7, 8]. This is most often seen in pneumonia [9]. However, hyponatremia also is a known marker of spontaneous bacterial peritonitis in liver cirrhosis [10]. 

Peritonitis can lead to increased vagal tone [11] leading to consecutive ST-elevation in electrocardiogram, sometimes mimicking an acute myocardial infarction [12]. Due to age-related prevalence of coronary heart disease, only patients older than 50 years have a routine ECG done at our institution.

We aimed to evaluate symptoms and signs (pain score, heart rate, blood pressure, and body temperature), laboratory values (CRP, leukocytes, hematocrit, sodium, and potassium), and ECG signs of increased vagal tone (symmetroid high T-waves, and AV blocks) as markers of infectious colon perforation in patients older than 50 years.

## 2. Materials and Methods

All patients older than fifty years with sigmoid diverticulitis or appendicitis admitted to our hospital between January 2008 and December 2010 were included in this study. Patients who were fifty years old or younger were excluded from the study. Patient identification was done by screening all radiology reports from this period with the search term “diverti*” and by screening all operation reports from this period with the search term “append*.” The presence of perforation was diagnosed by computer tomography (extraintestinal air, extraintestinal feces, abscess formation, or enterovesical fistula) and/or by surgical exploration. 

Finally, two hundred and eighty-two consecutive patients older than fifty years with sigmoid diverticulitis (*n* = 198) or with acute appendicitis (*n* = 84) were retrospectively analyzed.

At admission to the emergency station, pain score was assessed using a scale from 0 (no pain at all) to 10 (most severe pain), heart rate and blood pressure were measured using an automatic blood pressure machine, and body temperature was measured with a tympanic thermometer. Blood samples were taken before starting any infusion therapy in order to do a blood count and to analyze CRP, the electrolytes, and creatinine levels. Then a 12-lead ECG was done routinely.

Normal CRP values were recorded as below 5 mg/L; for the analyses, we set the negative results to 2.5 mg/L in order to prevent a bias. The threshold for elevated leukocytes was set to >10/nL and the threshold for hyponatremia was set to <136 mmol/L. Vasovagal signs in ECG were defined as symmetroid T-waves longer than two-thirds of the R-wave in any ECG lead or presence of AV block.

Stata 10.0 for Windows was used for computing statistical tests. The category data were summarized and a two-tailed Fisher's test was performed. The quantitative data was compared using the Wilcoxon rank sum test. A logistic regression analysis was performed to compare the diagnostic values of those markers that correlated significantly with colon perforation in univariate analysis. The study has been reviewed by the Ethics committeeof Basel (Ref.-Nr. 25/13). 

## 3. Results and Discussion

**Figure 1:**
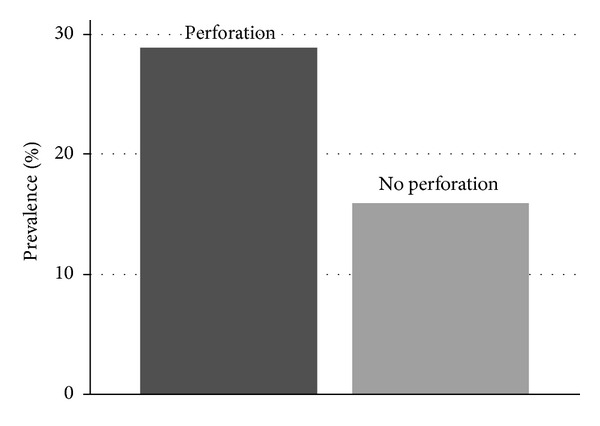
Hyponatremia in infectious colonic perforation. The prevalence of hyponatremia (<136 mmol/L) in the group with and in the group without colon perforation in infectious colonic diseases (*P* = 0.013, two-tailed Fisher's exact test).

One hundred and three patients were classified into the group with infectious colon perforation and one hundred and seventy-nine patients into the group without (perforation rate 37%). The group with and the group without infectious colon perforation were comparable with regard to baseline data and to regular medicament intake (Table 1). In univariate analysis, fever (>37.5°C), body temperature (*P* < 0.001), tachycardia (>100 bpm), heart rate, hyponatremia (<136 mmol/L), sodium, markedly elevated CRP (>50 mg/L), leukocytosis (>10/nL), and leukocytes correlated with infectious colon perforation, while signs in ECG, pain score, systolic blood pressure, hematocrit, creatinine, and potassium did not. The prevalence or mean values and the corresponding *P* values of the assessed markers of infectious colon perforation are shown in Table 2. In logistic regression analysis, only CRP (*P* < 0.001, OR  =  1.006), body temperature (*P* = 0.029, OR  =  1.508), and sodium (*P* = 0.047, OR  =  0.912) correlated significantly with infectious colon perforation, while heart rate (*P* = 0.404, OR  =  1.007) and leukocytes (*P* = 0.069, OR = 1.054) did not. The significant difference of prevalence of hyponatremia in the group with and in the group without colon perforation is shown in Figure 1.  Table 3 shows the specificities, sensitivities, and likelihood ratios of the markers that correlated significantly with colon perforation.

Hyponatremia, tachycardia, and fever have a moderate-to-high specificity for colon perforation. Therefore, their presence should raise suspicion of perforation. However, as the sensitivity of these three markers is poor, their absence cannot predict the absence of colon perforation in appendicitis or in sigmoid diverticulitis. 

The sensitivity of markedly elevated CRP and leukocytosis is moderate, while the specificity is poor. Therefore, the absence of these markers can be used to exclude colon perforation in appendicitis or in sigmoid diverticulitis. However, if present, they are not strong predictors of colon perforation in appendicitis or in sigmoid diverticulitis.

To our knowledge, no other study has found hyponatremia to be correlated significantly with colon perforation with consecutive peritonitis in appendicitis or sigmoid diverticulitis. This is of certain clinical importance as sodium measurement costs much less than measurement of CRP or other inflammation markers and sodium is often measured in the daily routine.

Limitations such as possible patient selection, missing data, and confounding are possible due to the retrospective nature of this study. However, the results are strengthened by the fact that all consecutive patients older than fifty years with appendicitis or with acute sigmoid diverticulitis admitted between January 2008 and December 2010 to one hospital were included in this study and that pain score, heart rate, blood pressure, body temperature, and laboratory values were measured before any therapy began and later statistically assessed.

## 4. Conclusion

As well as body temperature and CRP level, hyponatremia is a relevant and specific marker of infectious colon perforation in patients older than fifty years.

## Figures and Tables

**Table 1:** Baseline data and regular medicament intake of the group with and the group without infectious colon perforation.

	Colon perforation (*n* = 103)	No colon perforation (*n* = 179)	*P* value
Male gender	57%	50%	*P* = 0.265^a^
Mean age (standard deviation)	66.6 years (11.1)	66.7 years (10.7)	*P* = 0.923^b^
b-blocker	24%	18%	*P* = 0.274^a^
ACE/AT blocker	27%	28%	*P* = 0.891^a^
Calcium blocker	5%	7%	*P* = 0.611^a^
Diuretics	14%	13%	*P* = 0.853^a^
Digoxin	2%	1%	*P* = 0.626^a^

^a^Two-tailed Fisher's exact test; ^b^Wilcoxon rank sum test.

**Table 2:** Prevalence and mean values (standard deviation) of markers of infectious colon perforation.

	Colon perforation (*n* = 103)	No colon perforation (*n* = 179)	*P* < 0.001^b^
Fever (>37.5°C)	28%	13%	*P* = 0.002^a^
Body temperature (°C)	37.2 (0.8)	36.8 (0.7)	*P* < 0.001^b^
Tachycardia (>100 bpm)	21%	9%	*P* = 0.006^a^
Heart rate (bpm)	87.2 (20.5)	80.7 (15.0)	*P* = 0.005^b^
Hyponatremia (<136 mmol/L)	29%	16%	*P* = 0.013^a^
Sodium (mmol/L)	136.9 (3.3)	138.4 (3.0)	*P* < 0.001^b^
Markedly elevated CRP (>50 mg/L)	80%	60%	*P* = 0.001^a^
CRP (mg/L)	131.3 (91.1)	85.1 (68.7)	*P* < 0.001^b^
Symmetroid high T-waves (>2/3 R-wave)	13%	11%	*P* = 0.671^a^
AV blocks	1.1%	4.4%	*P* = 0.257^a^
Pain score (1–10)	4.4 (3.0)	3.9 (2.8)	*P* = 0.436^b^
Systolic blood pressure (mm Hg)	136.0 (21.5)	139.1 (21.7)	*P* = 0.278^b^
Hematocrit (%)	42.7 (4.6)	41.7 (4.6)	*P* = 0.090^b^
Potassium (mmol/L)	4.2 (0.40)	3.9 (0.40)	*P* = 0.175^b^
Creatinine (umol/L)	84.7 (37.8)	79.4 (28.2)	*P* = 0.283^b^

^a^Two-tailed Fisher's exact test; ^b^Wilcoxon rank sum test; bpm: beats per minute; CRP: C-reactive protein.

**Table 3:** Specificities, sensitivities, percentage of correctly classified patients, and the likelihood ratios of the markers correlating significantly with infectious colon perforation.

Marker	Sensitivity	Specificity	Correctly classified	Likelihood ratio +	Likelihood ratio −
Fever (>37.5°C)	28%	87%	66%	2.2	0.8
Tachycardia (>100 bpm)	21%	91%	65%	2.4	0.9
Hyponatremia (<136 mmol/L)	31%	79%	61%	1.5	0.9
Markedly elevated CRP (>50 mg/L)	80%	40%	54%	1.3	0.5
Leukocytosis (>10/nL)	74%	39%	51%	1.2	0.7
